# The immunologic tumor microenvironment in endometrioid endometrial cancer in the morphomolecular context: mutual correlations and prognostic impact depending on molecular alterations

**DOI:** 10.1007/s00262-020-02813-3

**Published:** 2020-12-19

**Authors:** Barbara Willvonseder, Fabian Stögbauer, Katja Steiger, Moritz Jesinghaus, Peer-Hendrik Kuhn, Christine Brambs, Jutta Engel, Holger Bronger, Georg Philipp Schmidt, Bernhard Haller, Wilko Weichert, Gisela Keller, Aurelia Noske, Nicole Pfarr, Melanie Boxberg

**Affiliations:** 1 Institute of Pathology, Technical University Munich, Trogerstrasse 18, 81675 Munich, Germany; 2 German Cancer Consortium (DKTK), Partner Site Munich, Munich, Germany; 3 German Cancer Consortium (DKTK), Partner Site Munich, Institute for Translational Cancer Research, Munich, Germany; 4 Department of Obstetrics and Gynecology, Technical University of Munich, Munich, Germany; 5 Munich Cancer Registry, Institute for Medical Information Processing, Biometry and Epidemiology (IBE), Ludwig-Maximilians-University (LMU), Munich, Germany; 6 Institute for Epidemiology and Statistics, Technical University Munich, Munich, Germany

**Keywords:** Endometrioid endometrial cancer, Molecular subgroups, Immunologic microenvironment, Prognostic impact

## Abstract

**Objective:**

POLE-mutant, microsatellite-instable (MSI), p53-mutant and non-specific molecular profile (NSMP) are TCGA-defined molecular subgroups of endometrial cancer (EC). Hypothesizing that morphology and tumor immunology might differ depending on molecular background concerning composition and prognostic impact, we aimed to comprehensively interconnect morphologic, immunologic and molecular data.

**Methods:**

TCGA-defined molecular groups were determined by immunohistochemistry and sequencing in *n* = 142 endometrioid EC. WHO-defined histopathological grading was performed. The immunologic microenvironment (iTME) was characterised by the quantification of intraepithelial and stromal populations of tumor-infiltrating lymphocytes (TIL: overall T-cells; T-Killer cells; regulatory T-cells (Treg)). Immunologic parameters were correlated with WHO-grading, TCGA-subgroups and prognosis.

**Results:**

High density TIL were significantly more frequent in high-grade (G3) compared to low-grade (G1/2) EC in the whole cohort and in the subgroup of POLE-wildtype-/microsatellite-stable-EC. MSI was associated with high-level TIL-infiltration when taking into account the type of mismatch repair defect (MLH1/PMS2; MSH2/MSH6). Prognostic impact of biomarkers depended on molecular subgroups: In p53-mutant EC, Treg were independently prognostic, in NSMP, the unique independently prognostic biomarker was WHO-grading.

**Conclusions:**

EC morphology and immunology differ depending on genetics. Our study delineated two molecularly distinct subgroups of immunogenic EC characterized by high-density TIL-infiltration: MSI EC and high-grade POLE-wildtype/microsatellite-stable-EC. Prognostic impact of TIL-populations relied on TCGA-subgroups indicating specific roles for TIL depending on molecular background. In NSMP, histopathological grading was the only prognostic biomarker demonstrating the relevance of WHO-grading in an era of molecular subtyping.

**Supplementary Information:**

The online version contains supplementary material available at 10.1007/s00262-020-02813-3.

## Introduction

Endometrial cancer (EC) is the most common gynaecologic malignancy with an estimated number of over 60.000 newly diagnosed cases in the United States accounting for 12.000 deaths in 2019. Incidence and mortality have been rising over the last years [1].

The World Health Organisation (WHO) Classification of Tumours of the Female Reproductive Organs [2] classifies EC into histologic subtypes (e.g. endometrioid; serous; clear cell) and defines a histopathologic grading of the endometrioid subtype into G1, G2 (low-grade) and G3 (high-grade; reviewed in [3, 4]) based on the morphologic features glandular vs. solid growth. This classical view has been complemented by insights into molecular genetics. “The Cancer Genome Atlas” (TCGA) published a comprehensive molecular characterisation based on exome sequencing of EC resulting in a classification into four genetically defined subgroups [5]: (1) Polymerase-Ɛ (POLE) ultramutated cases, (2) Microsatellite instable (MSI) cases with defective mismatch repair (MMR) due to the loss of function of MMR proteins MLH1/PMS2 or MSH2/MSH6, (3) Copy number high cases (“serous-like”), characterized by frequent mutations in TP53, extensive copy number variation, low mutational rate and (4) Copy number low microsatellite stable (MSS) cases with no defining molecular alteration (Non-specific molecular profile; NSMP). Subsequently the TCGA classification was reliably reproduced by two classifiers which are available for routine pathological analysis referred to as ProMisE/Vancouver and PORTEC/Leiden based on the following analysis: POLE mutational analysis by polymerase chain reaction (PCR) followed by mismatch repair protein immunohistochemistry (MMR) and p53 immunohistochemistry [3, 6–8].

The clinical outcome of EC patients varies significantly, suggesting a biological diversity of EC that is not fully reflected in the current models [9]. It is well-recognized that POLE-mutant cases carry an extremely favourable prognosis whereas copy number high cases show an unfavourable outcome necessitating aggressive treatment (reviewed in [3, 10]). However, prognostic biomarkers for MSI and NSMP cases remain to be elucidated as these patients show varying clinical courses with an overall intermediate prognosis [5, 10]. Therefore, treatment stratification to avoid under- and overtreatment of patients is difficult, especially in EC with MSI or NSMP. Furthermore, it is unclear if WHO-grading can provide additional information for treatment decisions as the prognostic value of WHO grading in the context of the novel molecular data is not yet elucidated.

Not only knowledge on genetics but as well on tumor immunology has progressed. Comprehensive studies in various tumor entities revealed that a high mutational load with subsequent high numbers of immunogenic neoantigens leads to a strong anti-tumoral cytotoxic T-cell response [11, 12]. In general, based on the presence or absence of T-cells, characterized by expression of CD3 (reviewed in [13]), the immunologic tumor microenvironment (iTME) is classified as “T-cell inflamed” versus “non T-cell inflamed” [14, 15]. Several subsets of T-cells play—partially antagonising—roles in the immunologic host response: CD8 + tumor-infiltrating lymphocytes (TIL; T-Killer cells) are crucial for a potent cytotoxic antitumor response [14, 15]. Regulatory T-cells (Treg), specifically characterized by the expression of the transcription factor FoxP3 [16], are involved in the tumor-host interaction by suppression of the immune responses [4, 17]. In EC, the iTME is—in accordance with the above described mechanisms—composed of several populations of immune cells. Increased numbers of CD3 + TIL have been associated with a favourable [18], FoxP3 + Tregs with a poor outcome [19]. However, studies comprehensively incorporating morphologic, molecular and immunologic data are still rare [12, 20–23] with only few studies comprising comprehensive data on TCGA-defined molecular subgroups.

The overriding hypothesis of our study was, that the role of EC morphology represented by WHO grading as well as influence and infiltration patterns of various TIL populations might differ depending on molecular background (POLE/P53 mutational status; Microsatellite status). Morphology and iTME might therewith carry differing prognostic impact depending on TCGA grouping of EC and might have the potential to be prognostic biomarkers for specific molecular defined EC subgroups (e.g. MSI and NSMP EC). Targeting these hypotheses, we analysed the immune contexture in a morphologically and molecular well-characterized homogeneous cohort of primary, untreated endometrioid EC. We correlated immunologic with morphologic data and TCGA-subgroups and analysed the prognostic impact of morphologic and immunologic biomarkers in the whole patient cohort and in the context of the TCGA-subgroups. With this study, we present a comprehensive analysis of morphomolecular and immunologic data in endometrioid EC.

## Material and Methods

### Patient cohort and clinico-pathological data

Our cohort included *n* = 142 therapy-naïve patients diagnosed with endometrioid EC between 2000 and 2014 who underwent resection of primary tumors at Klinikum Rechts der Isar, Technical University of Munich, Germany. Grading was undertaken according to the current WHO classification of tumors of the female reproductive organs [2]. Staging was performed according to the UICC/FIGO tumor, node and metastasis classification (7th edition; 2011)[24]. Patients received standardized adjuvant treatment and follow-up according to German guidelines [25]. Median follow-up time of patients alive (88/142; 62.0%) was 63.0 months (12.0–191.0 months), median follow-up of deceased patients (54/142; 38.0%) was 33.0 months (1.0–141.0 months), for the whole patient cohort, median follow-up was 74.0 (5.7) months. Detailed clinico-pathological data are given in Table 1. Approval for the study was obtained from the Ethics Review Committee of the Technical University of Munich (331/17).

**Table 1:** Clinicopathological and molecular data

	Number of cases	Percentage of cases (%)
Median age (range)		
69.7 (36.7–94.0)		
pT stage		
1a/1b	101	71.1
2	24	16.9
3	16	11.3
N/A	1	0.7
pN stage		
0	132	93.0
1	10	7.0
M stage		
0	135	95.1
1	7	4.9
FIGO Stage		
Ia/Ib	100	70.4
II	19	13.4
III	16	11.3
IV	7	4.9
Grading		
1	45	31.7
2	45	31.7
3	52	36.6
p53 status		
p53 mutation	20	14.1
p53 wildtype	122	85.9
Microsatellite status		
MSS	93	65.5
MSI (MLH1/PMS2)	42	29.6
MSI (MSH2/MSH6)	7	4.9
POLE status		
POLE mutation	7	4.9
POLE wildtype	135	95.1
Molecular Classification		
POLE-mutant	7	4.9
MSI	47	33.1
P53-mutant	12	8.5
NSMP	76	53.5

### Tissue micro array construction

Formalin-fixed paraffin-embedded tumor samples were assembled into tissue micro arrays (TMA) using a Tissue Microarrayer (Beecher Instruments) with a core size of 0.6 mm. Three cores per case, one from the invasion front and two from tumor core region, were selected from the primary tumors after reviewing the whole tumor slide. Areas were marked taking into account TIL infiltration and were representative for TIL distribution of the whole tumors.

### Immunohistochemistry (IHC)

An automated immunostainer (Ventana Benchmark XT) with an ultraView DAB detection kit (Ventana Medical Systems, Roche) was used for immunohistochemical staining of 2 µm sections from the TMA. Primary antibodies against p53 (clone DO-7, dilution 1:200, Dako), MSH2 (clone G219-1129, dilution 1:200, Cell Marque), MSH6 (clone 44, dilution 1:400, BD Transduction Laboratories), PMS2 (clone EPR3974, ready to use, Roche), MLH1 (clone M1, ready to use, Ventana), CD3 (clone MRQ-39, dilution 1:500, Cell Marque), CD8 (clone C8/144B, dilution 1:100, Dako) and FoxP3 (clone 236A/E7, dilution 1:200, Abcam) were applied. Appropriate positive and negative controls were run in parallel.

### Scoring of immunohistochemical stainings

**Fig. 1:**
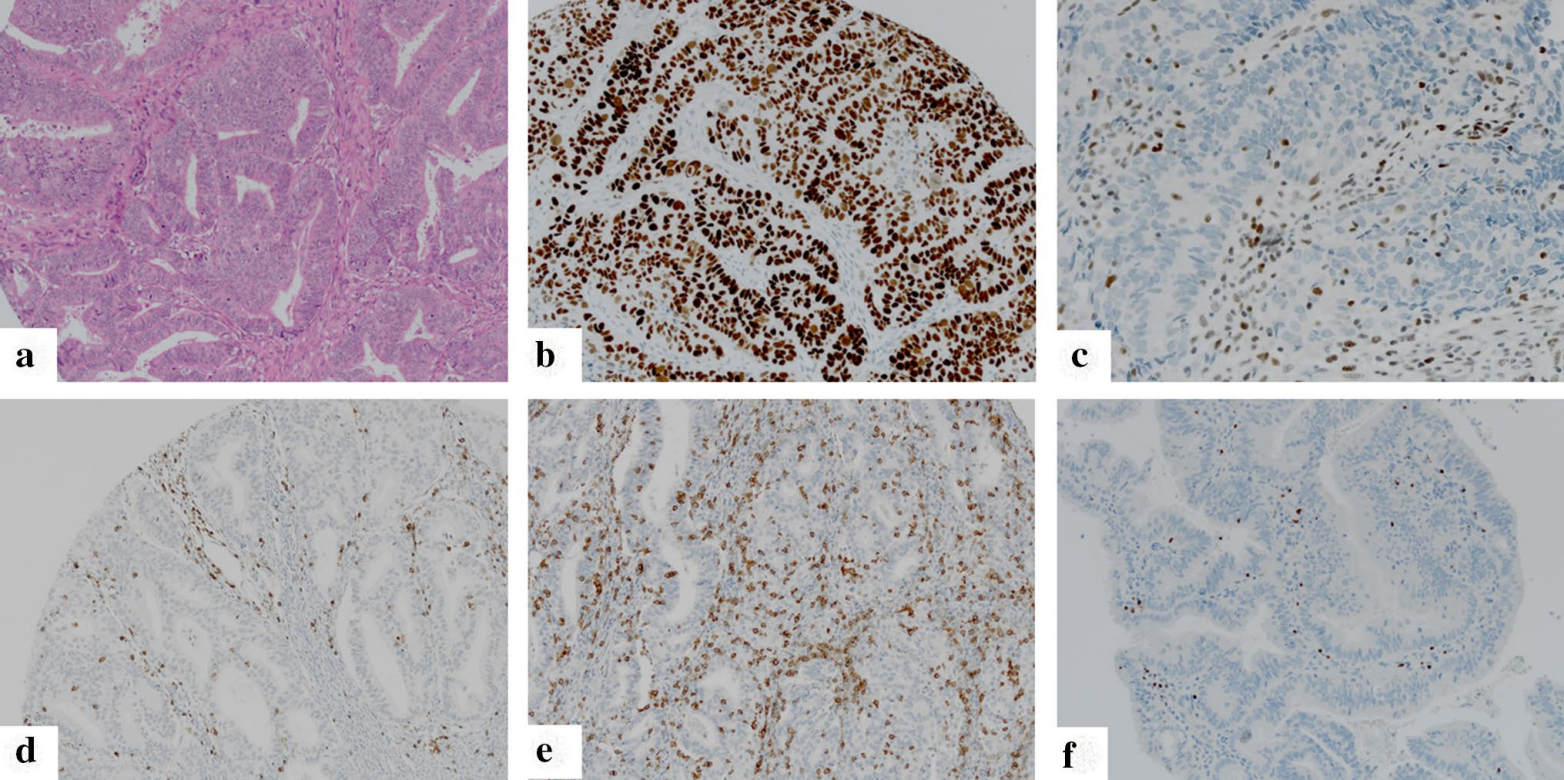
Representative micrographs of endometrioid endometrial carcinoma: **a** hematoxylin–eosin staining; **b** p53 staining showing a p53-mutant carcinoma with immunohistochemical staining score 2; **c** MLH1 staining showing a carcinoma with loss of MLH1 expression (positive internal control); **d** CD3 staining visualizing CD3 + Pan T-cell infiltrate; **e**; CD8 staining visualizing CD8 + T Killer-cell infiltrate (**f**) FoxP3 staining with nuclear positivity in regulatory T-cells

Testing of microsatellite status as well as analysis of p53 status were performed by immunohistochemistry in analogy to ProMisE/Vancouver and PORTEC/Leiden classifiers [3, 6, 8] taking into account all tumor cores. MSI was determined as two markers negative (MSH2/MSH6; MLH1/PMS2). IHC score 0 (complete p53 negativity) or IHC score 2 (strong nuclear p53 positivity in all tumor cells) was used as a surrogate marker for p53-mutation. Intermediate/heterogeneous nuclear staining (score 1) was documented as p53 wild type (Fig. 1, Supplementary Fig. 1).

Staining results of p53, MSH2, MSH6, MLH1 and PMS2 stains on TMA which showed staining results close to respective cut-offs were repeated on whole slides of full tissue blocks to reach a final result for respective stainings.

For analyses of TIL populations (Fig. 1; Supplementary Fig. 1), each tumor core was evaluated separately and the average density across all cores of specific regions (invasive front; tumor center) and whole tumor was calculated. The analysis of the TIL subpopulations was performed in three areas: (1) *Intraepithelial TIL*: the tumor region of the respective cores showing the highest density of the TIL population was selected on low power magnification (4x). Within this region, the amount of intraepithelial TIL (CD3i; CD8i; FoxP3i) was scored by counting the absolute number of TIL within 100 tumor cells using high power magnification (40x [26]). (2) *Stromal TIL*: In analogy to previous TIL-scoring approaches [27] density was evaluated by determination of percentage of the tumor stroma occupied by the respective TIL populations (CD3s; CD8s; FoxP3s). (3) *Overall TIL:* Density was scored via determination of the percentage of the tumor area (exclusion of necrosis) occupied by the respective TIL population (CD3o; CD8o; FoxP3o) [28].

Absolute values for TIL infiltrates were documented and subsequently assigned to scoring groups as described below.

### Cut-off determination for TIL infiltration

Using disease-specific survival (DSS) as an endpoint for the determination of the optimal prognostic cut-off values, receiver operating characteristic (ROC) curves were calculated for TIL density and followed by area under the curve (AUC) analysis. Cut-offs for high versus low TIL density were set at values with the highest Youden's index (Supplementary Table 1).

### DNA extraction

After marking of the tumor area and annotation of percentage of vital tumor tissue (≥ 50% tumor cell content) for micro-dissection, DNA was extracted using the Maxwell 16 RSC extraction system (Promega) according to the manufacturer´s protocols. DNA concentration was measured fluorometrically using the QuBit 3.0 system (Thermo Fisher Scientific) and DNA quality was determined by a qPCR assay (RNAseP assay, Thermo Fisher Scientific).

### *POLE* (NM.006231) mutational analysis

All cases were analyzed for POLE-Ɛ-mutations by Sanger sequencing of exons 9, 11, 13, and 14, which were amplified using primers as previously described [29]. Subsequent Sanger sequencing was conducted on a 3130 genetic analyzer (Applied Biosystems) using 5 µl amplified DNA/sample and the BigDye Terminator Cycle Sequencing Kit (Applied Biosystems) according to the manufacturers` protocols. Reported files were examined using BioEdit version 7.2.5.

### Statistics

Analyses were performed using SPSS 25 (SPSS Inc.) and R 3.6.1. The distribution of qualitative data was compared between groups using *χ*^2^-test or Fisher’s exact test. Survival probabilities were plotted with the cumulative incidence function. Median follow-up was estimated with the Kaplan–Meier estimate for potential follow-up, mean follow-up and 5 year survival by Kaplan Meier estimate. Overall survival (OS) was defined as patients alive at the end of follow-up, disease-specific survival (DSS) as all patients, who did not suffer from disease-related death. Disease-free survival (DFS) included all patients, who did not suffer from disease progression/recurrence during follow-up. Multivariate survival analysis was performed with the Cox Proportional Hazard model. All statistical tests were performed on exploratory two-sided 5% significance level.

## Results

### Morphologic and molecular characterization of EC

90/142 (63.4%) EC were classified as low grade (G1/G2), 52 (36.6%) as high grade (G3). According to the above described classifiers [3, 6–8], the study contained 4.9% POLE-mutant (detailed list of mutations in Supplementary Table 2), 33.1% MSI, 8.5% p53-mutant and 53.5% NSMP cases (Table 1).

### Composition of populations of tumor infiltrating lymphocytes

Absolute infiltration densities (mean; median; range) of TIL populations are given in Supplementary Table 3. Classification in scoring groups showed the following results (o = overall; i = intraepithelial; s = stromal): CD3i^high^ 112/142 (78.9%); CD3s^high^ 79/142 (55.6%); CD3o^high^ 51/142 (35.9%). CD8i^high^ 71/142 (50.0%); CD8s^high^ 103/142 (72.6%); CD8o^high^ 81/142 (57.0%). FoxP3i^high^ 5/142 (3.5%); FoxP3s^high^ 29/142 (20.4%); FoxP3o^high^ 28/142 (19.7%) (Supplementary Table 4; Supplementary Table 5). High density immune cell infiltrate of all analyzed TIL populations was significantly more frequent at the invasive front compared to tumor center (*p* < 0.01).

### Correlation of immunologic variables with morphomolecular and clinical data

**Fig. 2:**
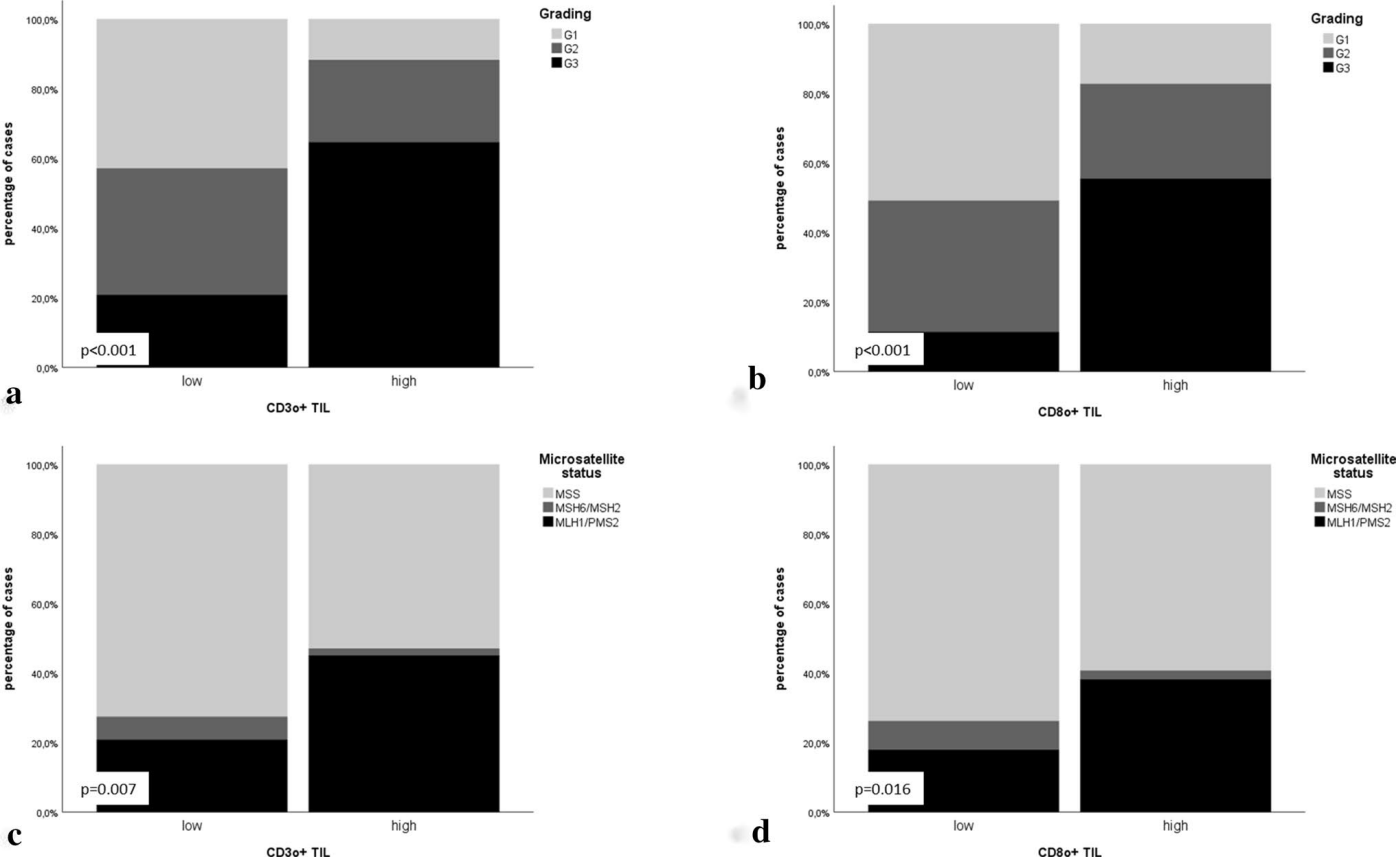
Association of CD3o + and CD8o + TIL infiltrates (overall infiltration density) with morphomolecular parameters: **a** CD3o + TIL infiltrate correlated with grading; **b** CD8o + TIL infiltrate correlated with grading; **c** CD3o + TIL infiltrate correlated with microsatellite status; **d** CD8o + TIL infiltrate correlated with microsatellite status

Immunologic-morphologic correlation: A high-density Pan T-cell infiltrate (CD3 +) and T-Killer cell infiltrate (CD8 +) was significantly more frequent in high-grade EC whereas in contrast a high density Treg infiltrate was more frequently observed in low-grade cases (*p* < 0.05; Fig. 2; detailed numbers and *p* values in Table 2, Supplementary Table 4).

**Table 2:** Correlation of immunologic variables (overall TIL) with morphomolecular data

	Grading (WHO)							Grading (WHO)					p53 status					Microsatellite status					Microsatellite status							POLE status				
	G1		G2		G3			Low grade (g1/g2)		High grade (g3)			Mutated		Wildtype			MSI		MSS			MLH1/PMS2 loss		MSH2/MSH6 loss		MSS			Wildtype		Mutated		
	*n*	%	*n*	%	*n*	%	*p *value	n	%	*n*	%	*p *value	*n*	%	*n*	%	*p *value	*n*	%	*n*	%	*p *value	*n*	%	*n*	%	*n*	%	*p *value	*n*	%	*n*	%	*p *value
Overall TIL (CD3)																																		
Low	39	27.5	33	23.2	19	13.4	< 0.001	72	50.7	19	13.4	< 0.001	16	11.3	75	52.8	0.109	25	17.6	66	46.5	0.019	19	13.4	6	4.2	66	46.5	0.007	88	62.0	3	2.1	*0.230*
High	6	4.2	12	8.5	33	23.2		18	12.7	33	23.2		4	2.8	47	33.1		24	16.9	27	19.0		23	16.2	1	0.7	27	19.0		47	33.1	4	2.8	
Overall T-Killer cells (CD8)																																		
Low	31	21.8	23	16.2	7	4.9	< 0.001	54	38.0	7	4.9	< 0.001	11	7.7	50	35.2	0.241	16	11.3	45	31.7	0.072	11	7.7	5	3.5	45	31.7	0.016	59	41.5	2	1.4	*0.430*
High	14	9.9	22	15.5	45	31.7		36	25.4	45	31.7		9	6.3	72	50.7		33	23.2	48	33.8		31	21.8	2	1.4	48	33.8		76	53.5	5	3.5	
Overall regulatory T-cells (FoxP3)																																		
Low	45	31.7	38	26.8	31	21.8	< 0.001	83	58.5	31	21.8	< 0.001	19	13.4	95	66.9	0.074	34	23.9	80	56.3	0.018	27	19.0	7	4.9	80	56.3	0.005	109	76.8	5	3.5	*0.546*
High	0	0.0	7	4.9	21	14.8		7	4.9	21	14.8		1	0.7	27	19.0		15	10.6	13	9.2		15	10.6	0	0.0	13	9.2		26	18.3	2	1.4	

Immunologic-molecular correlations: MSI was associated with CD3 + , CD8 + and FoxP3 + T-cell infiltrate when considering the specific mismatch repair defect (MLH1/PMS2 versus MSH2/MSH6): In MLH1/PMS2 deficient EC significantly higher levels of CD3 + ; CD8 + and FoxP3 + TIL infiltrates were observed compared to MSH2/MSH6-deficient and MSS cases (Fig. 2). No significant association of p53 and POLE mutation with immunologic parameters was found, most likely due to only rare mutant cases (*p* < 0.05; detailed numbers and *p* values in Table 2, Supplementary Table 4).

Immunologic-clinical correlations: Correlation of immunologic with clinical data revealed only few significant associations. Amongst others, overall Treg (FoxP3o) density was significantly higher in cases with high FIGO stage (*p* < 0.05; detailed numbers and *p* values Supplementary Table 5).

### Correlation of immunologic variables with morphomolecular data in the molecular defined subgroup MSS and POLE wildtype EC

POLE-mutant and MSI EC are known to be highly immunogenic due to their high mutational load. We hypothesized that a further immunogenic subgroup of EC potentially exists among the subgroup of MSS EC without POLE mutation (POLE-wildtype + MSS EC). Therefore, we analyzed associations of immunologic and morphomolecular data in this specific molecular defined subgroup (*n* = 88): High-density immune cell infiltrate (CD3; CD8; FoxP3) was significantly correlated with high-grade (G3) morphology (*p* < 0.05; Supplementary Table 6).

### Survival associations of immunologic parameters in the whole patient cohort

Analysing survival associations in the whole patient cohort, we did not detect any significant correlations (Supplementary Table 7 summarizes OS, DSS and DFS for all immunologic variables).

### Survival associations of immunologic parameters depending on TCGA-defined subgroups

In line with previous literature, molecular classification of EC according to TCGA resulted in four prognostic groups (POLE-ultramutated; MSI; P53-mutant; NSMP) significantly associated with DSS (*p* = 0.041) and DFS (*p* = 0.014) (Supplementary Fig. 2) with POLE-mutant EC carrying the most favourable prognosis and p53-mutant EC showing the worst outcome. These data may prove the robustness of our results.

In p53-mutant EC, stromal Treg were an independent prognostic factor for OS (*p* = 0.035), DSS (*p* = 0.032) and DFS (*p* = 0.012). Median OS (DSS; DFS) of patients with FoxP3s^low^ was 83.0 (108.0; 90.0) months compared to 12.0 (12.0; 11.0) months of counterparts (FoxP3s^high^), 5 year survival rates were OS/DSS/DFS: 84.2%/84.2%/73.7% vs. 0.0%/0.0%/0.0%. Results of cox regression analysis (incorporating age, grading, FIGO stage) are given in Table 3.

**Table 3:** Multivariate statistical analysis for p53-mutant endometrial carcinoma (left side) and endometrial carcinoma with non-specific molecular profile (right side) under inclusion of age, grading, stage and FoxP3 + TIL (in p53-mutant endometrial carcinoma)

Endometrial carcinoma with p53 mutation					Endometrial carcinoma with non-specific molecuar profile (NSMP)				
	HR (OS)	Lower CI (95%)	Upper CI (95%)	*p* value		HR (DSS)	Lower CI (95%)	Upper CI (95%)	*p* value
Age					Age				
Per year	2.755	0.369	20.560	0.323	Per year	0.978	0.310	3.084	0.970
Grading					Grading				
1				0.698	Low				0.011
2	> 25	< 0.001	> 25		High	4.329	1.404	13.349	
3	> 25	< 0.001	> 25						
FIGO Stage					FIGO Stage				
Low (I/II)	12.477	0.682	> 25	0.089	Low (I/II)				0.019
High (III/IV)					High (III/IV)	4.375	1.270	15.069	
FoxP3 + TIL									
Low				0.035					
High	49.3	1.315	> 25						
	HR (DSS)	Lower CI (95%)	Upper CI (95%)	*p* value		HR (DFS)	Lower CI (95%)	Upper CI (95%)	*p* value
Age					Age				
Per year	1.791	0.134	23.978	0.660	Per year	1.572	0.561	4.401	0.390
Grading					Grading				
1				0.726	Low				0.042
2	> 25	< 0.001	> 25		High	2.778	1.037	7.442	
3	> 25	< 0.001	> 25						
FIGO Stage					FIGO Stage				
Low (I/II)				0.119	Low (I/II)				< 0.001
High (III/IV)	12.377	0.518	> 25		High (III/IV)	8.527	3.144	23.123	
FoxP3 + TIL									
Low				0.032					
High	71.588	1.430	> 25						
	HR (DFS)	Lower CI (95%)	Upper CI (95%)	*p* value					
Age									
Per year	0.477	0.050	4.506	0.518					
Grading									
1				0.991					
2	> 25	< 0.001	> 25						
3	> 25	< 0.001	> 25						
FIGO Stage									
Low (I/II)				0.029					
High (III/IV)	17.238	1.347	> 25						
FoxP3 + TIL									
Low				0.012					
High	147.060	2.976	> 25						

**Fig. 3:**
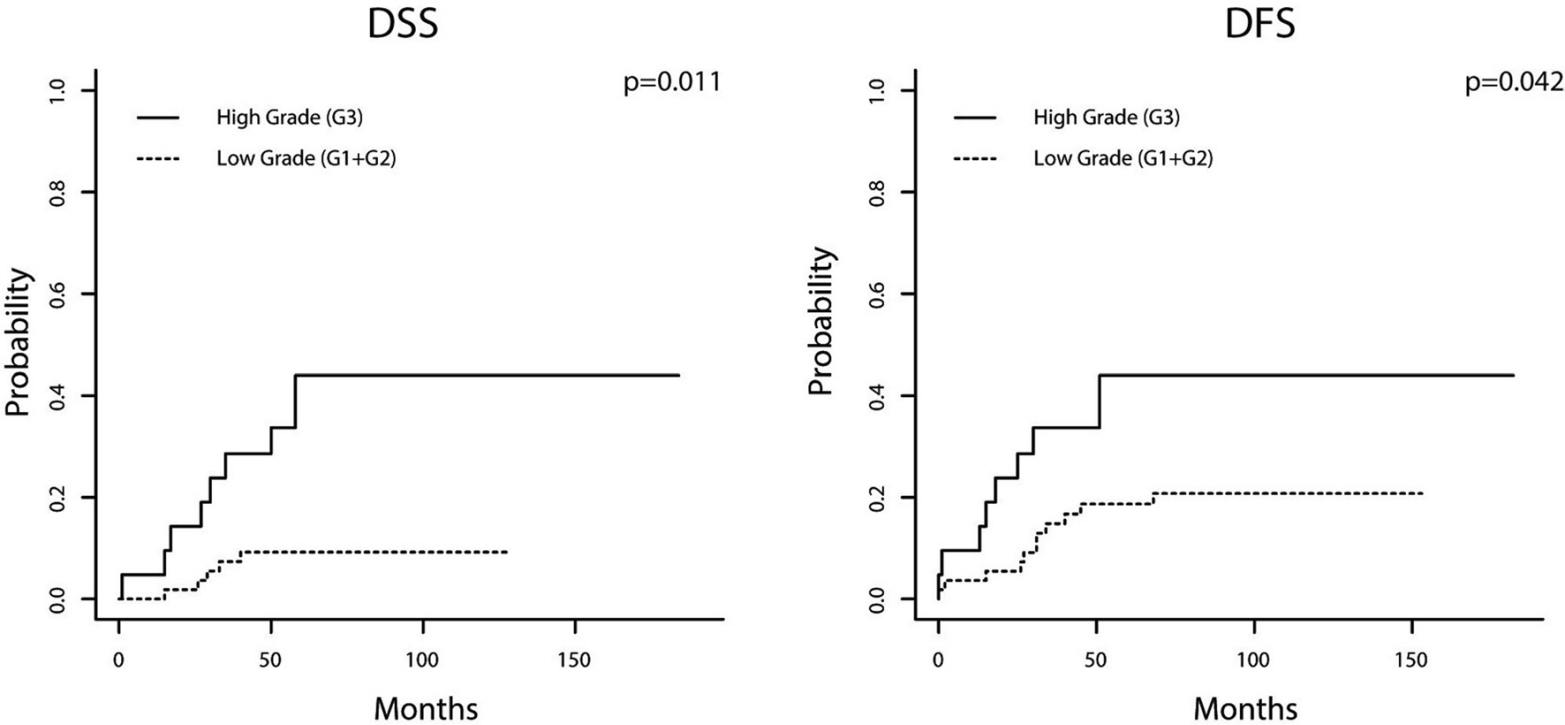
Cumulative incidence function visualizing disease-specific and disease-free survival depending on grading [carcinomas with non-specific molecular profile (**a**, **b**)]

In NSMP EC, WHO grading was identified as independent prognostic factor for DSS and DFS whereas immunologic variables did not influence outcome. Patients with low-grade EC had a mean DSS/DFS 117.7/127.1 months while mean DSS/DFS of counterparts with high-grade carcinomas was 107.8/112.9 months. 5 year survival rates were as follows: DSS 90.9%/57.1%; DFS 81.8%/57.1%. Resulting hazard ratios for high-grade EC were 4.2 for DSS (*p* = 0.11) and 2.8 for DFS (*p* = 0.042; Fig. 3; Table 3).

No correlation of immunologic parameters with outcome was observed in POLE-mutant cases.

## Discussion

EC is the malignancy with highest prevalence and incidence in gynaecology. Clinical outcome of patients varies significantly [9]—necessitating prognostic biomarkers to avoid over- or undertreatment. Molecular subgrouping as defined by TCGA into POLE-mutant EC, MSI EC, NSMP EC and p53-mutant EC renders valuable prognostic information with POLE-mutant cases carrying a favourable prognosis whereas P53-mutant EC show an unfavourable outcome necessitating aggressive treatment (reviewed in [3, 10]). However, prognostic biomarkers for MSI and NSMP cases remain to be elucidated as these patients show varying clinical courses with an overall intermediate prognosis [5, 10]. Furthermore, the future prognostic value of morphologic grading described by the WHO classification [3, 4] in the molecular context is up to date unclear as e.g. POLE-mutant EC show a high-grade (G3) morphology despite comparably benign clinical course [6, 7, 10]. TCGA-defined molecular groups might not only give prognostic information but may, furthermore, influence the composition and prognostic impact of the iTME of EC consisting of various TIL populations.

Aiming to gain data to further elucidate the role and impact of morphology and tumor immunology in the context of the underlying molecular alterations we analysed the immune contexture represented by the intratumoral T-cell infiltrate (Pan T-cells, T-Killer cells, Treg) in a morphologically and molecularly well-characterized homogeneous cohort of primary, untreated endometrioid EC. We correlated immunologic with morphologic data and TCGA-defined molecular subgroups and analysed the prognostic impact of morphologic and immunologic biomarkers in the whole patient cohort and in the context of TCGA-subgroups.

Confirming previous studies, our data show that subgroups of EC contain a T-cell inflamed immunogenic iTME represented by a dense T-cell infiltration [18, 20–22]. In accordance with our hypothesis outlined in the introduction, these immunogenic subgroups were characterized by distinct morphologic and molecular features:

We observed an immunogenic subgroup characterized by high-grade (G3) morphology: A high-density TIL infiltrate was significantly correlated with WHO-defined high-grade (G3) EC—a finding, which was observed as well in a previous analysis by Li et al. [30]. The correlation might, at first glance, be explained by a high number of high-grade morphology cases with MSI or POLE-mutation, both of which are known to show high-grade morphology and to be densely infiltrated by immune cells [17, 20, 22, 30–32]. But the correlation was found to be significant in the subgroup of MSS EC without POLE mutation (MSS/POLE wildtype EC), demonstrating, that an immunogenic subgroup of EC without MSI/POLE mutation is characterized by high-grade (G3) morphology.

Furthermore, MSI was confirmed to be a highly immunogenic subtype of EC: MSI EC were significantly associated with a high density TIL infiltrate. In previous studies these finding was linked to a high mutational load in MSI cancers [12, 20, 22, 23, 30, 31]. Interestingly, in our study, the correlation of MSI with high immunogenicity was only significant when the underlying type of MMR defect was included into calculation with MLH1/PMS2 defective cases being highly immunogenic in contrast to MSH2/MSH6 defective and MSS EC. This may give a hint that either type of MMR defect or sporadic versus hereditary origin of MSI might imply a very distinct iTME [33].

Interestingly, the inflammatory infiltrate showed heterogeneity considering their intratumoral distribution: the high density T-cell infiltration showed significantly higher levels within the invasive front.

Data concerning prognostic impact of immunologic biomarkers in EC in the context of TCGA-subgroups is up to date rare with previous studies including partially small cohorts with mixed EC subtypes and unclear genetic background [18, 19, 23, 30]. In the whole—molecularly highly heterogeneous—patient cohort, none of the analysed variables showed a prognostic impact. In contrast, when considering the molecular background, we found striking differences in the impact of certain T-cell populations on patient prognosis: In p53-mutant cases, Tregs showed a major prognostic impact. A high density Treg infiltrate was independently correlated with a poor patient prognosis. In NSMP EC, immunologic biomarkers revealed no prognostic impact—the only independent biomarker was histopathological WHO-based grading with high-grade EC carrying a significantly worse prognosis.

In accordance with our results, Yamagami et al. found Tregs to be a negative prognostic biomarker [19]. Previous results concerning further TIL populations were inconclusive: Yamashita et al. found an impact of CD8 + TIL on DFS [23] but only used univariate analysis. Cermakova identified CD3 + T-cell infiltrate to be prognostic but did not further subtype the TIL subpopulation [18]. These differing results may at least in part be explained by the fact that none of the previous studies analysed the prognostic impact of immunologic parameters in the context of TCGA subgroups.

The prognostic impact of Treg may be explained by the functional role of these lymphocyte populations in cancer immunity. Tregs suppress immune responses by suppression of activation, proliferation and effector functions of numerous cell types including T-Killer cells and are, therefore, involved in metastasis and progression [17]. In line with that, a high density Treg infiltrate may indicate a poor survival due to suppression of anti-cancer immune responses.

Taking together the results of our study the presented comprehensive analysis of morphology and iTME in the context of the TCGA-defined subgroups delineates major immunogenic EC subgroups: MSI EC, especially those with MLH1/PMS2 defect, and high-grade (G3) POLE-wildtype/MSS EC. We, furthermore, identify prognostic TIL subpopulations (Treg), which vary depending on TCGA subgroups indicating specific roles for TIL populations depending on molecular background. In NSMP EC, WHO grading was the only independent prognostic biomarker demonstrating the potential future relevance of WHO-grading in an era of molecular subtyping.

Our study clearly has some limitations. Potentially due to the low absolute number of cases with POLE mutation (which is nevertheless in accordance with the literature [34, 35]), we did not detect associations of POLE-mutant EC (which had been demonstrated in multiple previous studies) with T-cell infiltration. Furthermore, our study has a retrospective design necessitating prospective studies to confirm the findings.

## Supplementary Information

Below is the link to the electronic supplementary material.

Supplementary file1 (PDF 1262 KB)
