# Supplementary material for: The immunologic tumor microenvironment in endometrioid endometrial cancer in the morphomolecular context: mutual correlations and prognostic impact depending on molecular alterations
Source: Cancer Immunol Immunother. 2020 Dec 19;70(6):1679–89. doi: 10.1007/s00262-020-02813-3 (PMC8139910; doi:10.1007/s00262-020-02813-3)
Supplement: Supplementary file 1 — Supplementary file1 (PDF 1262 KB) [file 262_2020_2813_MOESM1_ESM.pdf]

	Coordinates of ROC curve					
Variable	positive if ≥	sensitivity	1 - specificity	specificity	sensitivity + specificity	Youden
Overall CD3+ TIL	-0,5000	1,000	1,000	0,000	1,000	0,000
	0,6250	0,967	0,948	0,052	1,018	0,018
	0,8750	0,900	0,866	0,134	1,034	0,034
	1,1250	0,867	0,835	0,165	1,032	0,032
	1,3750	0,833	0,814	0,186	1,019	0,019
	1,6250	0,833	0,794	0,206	1,040	0,040
	1,8750	0,800	0,784	0,216	1,016	0,016
	2,2500	0,733	0,753	0,247	0,981	-0,019
	2,6250	0,733	0,742	0,258	0,991	-0,009
	2,8750	0,700	0,742	0,258	0,958	-0,042
	3,2500	0,633	0,701	0,299	0,932	-0,068
	3,6250	0,633	0,670	0,330	0,963	-0,037
	3,8750	0,633	0,649	0,351	0,984	-0,016
	4,2500	0,600	0,619	0,381	0,981	-0,019
	4,7500	0,600	0,598	0,402	1,002	0,002
	5,2500	0,600	0,546	0,454	1,054	0,054
	5,7500	0,600	0,536	0,464	1,064	0,064
	6,2500	0,600	0,474	0,526	1,126	0,126
	6,7500	0,567	0,474	0,526	1,092	0,092
	7,2500	0,567	0,433	0,567	1,134	0,134
	7,6250	0,500	0,402	0,598	1,098	0,098
	8,1250	0,500	0,392	0,608	1,108	0,108
	8,7500	0,500	0,361	0,639	1,139	0,139
	9,5000	0,500	0,351	0,649	1,149	0,149
	10,1250	0,467	0,320	0,680	1,147	0,147
	10,6250	0,433	0,320	0,680	1,114	0,114
	11,7500	0,400	0,309	0,691	1,091	0,091
	13,7500	0,367	0,299	0,701	1,068	0,068
	15,5000	0,300	0,237	0,763	1,063	0,063
	16,7500	0,267	0,237	0,763	1,030	0,030
	18,7500	0,233	0,237	0,763	0,996	-0,004
	21,2500	0,133	0,196	0,804	0,937	-0,063
	23,7500	0,067	0,186	0,814	0,881	-0,119
	26,2500	0,067	0,144	0,856	0,922	-0,078
	28,7500	0,033	0,113	0,887	0,920	-0,080
	31,2500	0,000	0,103	0,897	0,897	-0,103
	33,7500	0,000	0,082	0,918	0,918	-0,082
	36,2500	0,000	0,072	0,928	0,928	-0,072
	38,7500	0,000	0,062	0,938	0,938	-0,062
	43,7500	0,000	0,052	0,948	0,948	-0,052
	48,7500	0,000	0,041	0,959	0,959	-0,041
	52,5000	0,000	0,031	0,969	0,969	-0,031
	60,0000	0,000	0,021	0,979	0,979	-0,021
	67,5000	0,000	0,010	0,990	0,990	-0,010
	71,0000	0,000	0,000	1,000	1,000	0,000
Overall CD8+ TIL	-1,0000	1,000	1,000	0,000	1,000	0,000
	0,1250	0,967	0,969	0,031	0,998	-0,002
	0,3750	0,967	0,959	0,041	1,008	0,008
	0,6250	0,900	0,856	0,144	1,044	0,044
	0,8750	0,867	0,794	0,206	1,073	0,073
	1,1250	0,800	0,722	0,278	1,078	0,078
	1,3750	0,767	0,701	0,299	1,066	0,066
	1,6250	0,767	0,660	0,340	1,107	0,107
	1,8750	0,767	0,639	0,361	1,127	0,127
	2,2500	0,767	0,546	0,454	1,220	0,220
	2,6250	0,600	0,546	0,454	1,054	0,054
	2,8750	0,600	0,536	0,464	1,064	0,064
	3,2500	0,600	0,505	0,495	1,095	0,095
	3,6250	0,600	0,464	0,536	1,136	0,136
	3,8750	0,533	0,464	0,536	1,069	0,069
	4,5000	0,467	0,412	0,588	1,054	0,054
	5,1250	0,467	0,381	0,619	1,085	0,085
	5,3750	0,433	0,371	0,629	1,062	0,062
	5,7500	0,400	0,371	0,629	1,029	0,029
	6,5000	0,367	0,361	0,639	1,006	0,006
	7,7500	0,300	0,320	0,680	0,980	-0,020
	9,2500	0,300	0,299	0,701	1,001	0,001
	10,5000	0,233	0,289	0,711	0,945	-0,055
	11,7500	0,200	0,278	0,722	0,922	-0,078
	13,0000	0,167	0,268	0,732	0,899	-0,101
	14,2500	0,167	0,247	0,753	0,919	-0,081
	15,5000	0,100	0,196	0,804	0,904	-0,096
	16,7500	0,100	0,175	0,825	0,925	-0,075
	18,7500	0,100	0,165	0,835	0,935	-0,065
	21,2500	0,033	0,144	0,856	0,889	-0,111
	23,7500	0,000	0,103	0,897	0,897	-0,103
	27,5000	0,000	0,052	0,948	0,948	-0,052
	31,2500	0,000	0,041	0,959	0,959	-0,041
	36,2500	0,000	0,031	0,969	0,969	-0,031
	47,5000	0,000	0,021	0,979	0,979	-0,021
	56,0000	0,000	0,000	1,000	1,000	0,000
Overall FoxP3+ TIL	-1,0000	1,000	1,000	0,000	1,000	0,000
	0,1250	0,933	0,979	0,021	0,954	-0,046
	0,3750	0,900	0,928	0,072	0,972	-0,028
	0,6250	0,700	0,732	0,268	0,968	-0,032
	0,8750	0,667	0,629	0,371	1,038	0,038
	1,1250	0,600	0,546	0,454	1,054	0,054
	1,3750	0,500	0,505	0,495	0,995	-0,005
	1,6250	0,433	0,495	0,505	0,938	-0,062
	1,8750	0,433	0,464	0,536	0,969	-0,031
	2,2500	0,367	0,381	0,619	0,985	-0,015
	2,7500	0,333	0,330	0,670	1,003	0,003
	3,2500	0,267	0,237	0,763	1,030	0,030
	3,7500	0,267	0,196	0,804	1,071	0,071
	4,2500	0,200	0,155	0,845	1,045	0,045
	4,7500	0,200	0,134	0,866	1,066	0,066
	5,5000	0,133	0,082	0,918	1,051	0,051
	6,2500	0,100	0,062	0,938	1,038	0,038
	6,7500	0,067	0,062	0,938	1,005	0,005
	7,2500	0,067	0,052	0,948	1,015	0,015
	8,0000	0,033	0,052	0,948	0,982	-0,018
	9,2500	0,033	0,031	0,969	1,002	0,002
	11,2500	0,000	0,031	0,969	0,969	-0,031
	13,7500	0,000	0,021	0,979	0,979	-0,021
	20,0000	0,000	0,010	0,990	0,990	-0,010
	26,0000	0,000	0,000	1,000	1,000	0,000
stromal CD3+ TIL	-0,5000	1,000	1,000	0,000	1,000	0,000
	0,6250	0,971	0,944	0,056	1,026	0,026
	0,8750	0,941	0,944	0,056	0,997	-0,003

	1,1250	0,941	0,935	0,065	1,006	0,006
	1,3750	0,941	0,926	0,074	1,015	0,015
	2,1250	0,941	0,917	0,083	1,025	0,025
	2,8750	0,941	0,889	0,111	1,052	0,052
	3,5000	0,912	0,880	0,120	1,032	0,032
	4,2500	0,912	0,861	0,139	1,051	0,051
	4,7500	0,912	0,852	0,148	1,060	0,060
	5,1250	0,853	0,833	0,167	1,020	0,020
	5,6250	0,853	0,824	0,176	1,029	0,029
	6,2500	0,853	0,815	0,185	1,038	0,038
	6,7500	0,824	0,815	0,185	1,009	0,009
	7,2500	0,794	0,778	0,222	1,016	0,016
	8,0000	0,794	0,769	0,231	1,026	0,026
	8,7500	0,794	0,741	0,259	1,053	0,053
	9,5000	0,765	0,741	0,259	1,024	0,024
	10,7500	0,706	0,731	0,269	0,974	-0,026
	12,0000	0,706	0,722	0,278	0,984	-0,016
	13,7500	0,706	0,685	0,315	1,021	0,021
	15,1250	0,706	0,667	0,333	1,039	0,039
	15,6250	0,706	0,657	0,343	1,048	0,048
	16,7500	0,706	0,648	0,352	1,058	0,058
	18,7500	0,706	0,630	0,370	1,076	0,076
	20,5000	0,706	0,602	0,398	1,104	0,104
	21,2500	0,706	0,593	0,407	1,113	0,113
	22,0000	0,706	0,574	0,426	1,132	0,132
	23,0000	0,647	0,556	0,444	1,092	0,092
	24,2500	0,647	0,537	0,463	1,110	0,110
	25,1250	0,647	0,528	0,472	1,119	0,119
	25,3750	0,588	0,519	0,481	1,070	0,070
	26,0000	0,588	0,509	0,491	1,079	0,079
	27,0000	0,588	0,500	0,500	1,088	0,088
	28,0000	0,588	0,472	0,528	1,116	0,116
	29,2500	0,559	0,472	0,528	1,087	0,087
	32,5000	0,441	0,463	0,537	0,978	-0,022
	36,2500	0,412	0,435	0,565	0,977	-0,023
	38,0000	0,382	0,417	0,583	0,966	-0,034
	39,2500	0,382	0,407	0,593	0,975	-0,025
	41,2500	0,324	0,343	0,657	0,981	-0,019
	43,7500	0,324	0,333	0,667	0,990	-0,010
	46,2500	0,265	0,315	0,685	0,950	-0,050
	48,7500	0,265	0,296	0,704	0,968	-0,032
	52,5000	0,206	0,222	0,778	0,984	-0,016
	56,2500	0,176	0,213	0,787	0,964	-0,036
	58,7500	0,176	0,204	0,796	0,973	-0,027
	62,5000	0,118	0,167	0,833	0,951	-0,049
	67,5000	0,118	0,130	0,870	0,988	-0,012
	72,5000	0,088	0,102	0,898	0,986	-0,014
	77,5000	0,029	0,074	0,926	0,955	-0,045
	83,7500	0,000	0,028	0,972	0,972	-0,028
	88,7500	0,000	0,019	0,981	0,981	-0,019
	92,5000	0,000	0,009	0,991	0,991	-0,009
	96,0000	0,000	0,000	1,000	1,000	0,000
epithelial CD3+ TIL	-0,5000	1,000	1,000	0,000	1,000	0,000
	0,7500	0,971	0,981	0,019	0,989	-0,011
	1,2500	0,912	0,870	0,130	1,041	0,041
	1,7500	0,882	0,759	0,241	1,123	0,123
	2,2500	0,765	0,667	0,333	1,098	0,098
	2,7500	0,676	0,620	0,380	1,056	0,056
	3,2500	0,618	0,556	0,444	1,062	0,062
	3,7500	0,588	0,546	0,454	1,042	0,042
	4,2500	0,500	0,500	0,500	1,000	0,000
	4,7500	0,500	0,481	0,519	1,019	0,019
	5,2500	0,500	0,426	0,574	1,074	0,074
	5,7500	0,471	0,407	0,593	1,063	0,063
	6,2500	0,441	0,352	0,648	1,089	0,089
	6,7500	0,441	0,343	0,657	1,099	0,099
	7,2500	0,382	0,315	0,685	1,068	0,068
	7,7500	0,353	0,278	0,722	1,075	0,075
	8,2500	0,353	0,241	0,759	1,112	0,112
	8,7500	0,294	0,222	0,778	1,072	0,072
	9,5000	0,176	0,222	0,778	0,954	-0,046
	10,2500	0,118	0,185	0,815	0,932	-0,068
	10,7500	0,118	0,176	0,824	0,942	-0,058
	11,5000	0,088	0,148	0,852	0,940	-0,060
	12,5000	0,059	0,139	0,861	0,920	-0,080
	13,5000	0,059	0,130	0,870	0,929	-0,071
	14,2500	0,059	0,102	0,898	0,957	-0,043
	15,2500	0,059	0,093	0,907	0,966	-0,034
	16,7500	0,029	0,093	0,907	0,937	-0,063
	19,0000	0,029	0,065	0,935	0,965	-0,035
	21,2500	0,029	0,056	0,944	0,974	-0,026
	22,7500	0,029	0,046	0,954	0,983	-0,017
	24,0000	0,029	0,037	0,963	0,992	-0,008
	24,7500	0,029	0,028	0,972	1,002	0,002
	30,5000	0,000	0,019	0,981	0,981	-0,019
	47,7500	0,000	0,009	0,991	0,991	-0,009
	60,5000	0,000	0,000	1,000	1,000	0,000
stromal CD8+ TIL	-1,0000	1,000	1,000	0,000	1,000	0,000
	0,1250	0,971	0,972	0,028	0,998	-0,002
	0,3750	0,971	0,954	0,046	1,017	0,017
	0,6250	0,912	0,880	0,120	1,032	0,032
	0,8750	0,912	0,861	0,139	1,051	0,051
	1,1250	0,882	0,843	0,157	1,040	0,040
	1,3750	0,853	0,824	0,176	1,029	0,029
	1,6250	0,853	0,815	0,185	1,038	0,038
	1,8750	0,853	0,806	0,194	1,047	0,047
	2,2500	0,853	0,713	0,287	1,140	0,140
	2,7500	0,853	0,685	0,315	1,168	0,168
	3,5000	0,735	0,620	0,380	1,115	0,115
	4,5000	0,706	0,593	0,407	1,113	0,113
	5,1250	0,618	0,556	0,444	1,062	0,062
	5,6250	0,618	0,546	0,454	1,071	0,071
	6,2500	0,618	0,537	0,463	1,081	0,081
	6,7500	0,588	0,509	0,491	1,079	0,079
	7,2500	0,588	0,491	0,509	1,097	0,097
	7,6250	0,559	0,472	0,528	1,087	0,087
	7,8750	0,559	0,463	0,537	1,096	0,096
	8,5000	0,559	0,454	0,546	1,105	0,105
	9,5000	0,559	0,435	0,565	1,124	0,124
	10,1250	0,529	0,407	0,593	1,122	0,122
	10,8750	0,500	0,398	0,602	1,102	0,102
	12,0000	0,471	0,398	0,602	1,072	0,072

	13,0000	0,471	0,389	0,611	1,082	0,082
	14,2500	0,412	0,380	0,620	1,032	0,032
	15,1250	0,353	0,370	0,630	0,983	-0,017
	15,8750	0,353	0,361	0,639	0,992	-0,008
	17,0000	0,324	0,361	0,639	0,962	-0,038
	18,7500	0,294	0,343	0,657	0,952	-0,048
	20,7500	0,235	0,324	0,676	0,911	-0,089
	22,0000	0,235	0,315	0,685	0,920	-0,080
	23,0000	0,235	0,306	0,694	0,930	-0,070
	24,2500	0,235	0,296	0,704	0,939	-0,061
	27,5000	0,206	0,241	0,759	0,965	-0,035
	31,7500	0,176	0,231	0,769	0,945	-0,055
	34,2500	0,176	0,222	0,778	0,954	-0,046
	37,5000	0,176	0,148	0,852	1,028	0,028
	41,2500	0,118	0,102	0,898	1,016	0,016
	43,7500	0,118	0,093	0,907	1,025	0,025
	47,5000	0,088	0,093	0,907	0,996	-0,004
	52,5000	0,029	0,056	0,944	0,974	-0,026
	57,5000	0,000	0,046	0,954	0,954	-0,046
	62,5000	0,000	0,037	0,963	0,963	-0,037
	70,0000	0,000	0,019	0,981	0,981	-0,019
	76,0000	0,000	0,000	1,000	1,000	0,000
epithelial CD8+ TIL	-1,0000	1,000	1,000	0,000	1,000	0,000
	0,2500	0,941	0,954	0,046	0,987	-0,013
	0,7500	0,882	0,917	0,083	0,966	-0,034
	1,2500	0,735	0,806	0,194	0,930	-0,070
	1,7500	0,676	0,704	0,296	0,973	-0,027
	2,2500	0,676	0,639	0,361	1,038	0,038
	2,7500	0,647	0,602	0,398	1,045	0,045
	3,2500	0,500	0,546	0,454	0,954	-0,046
	3,7500	0,441	0,519	0,481	0,923	-0,077
	4,2500	0,441	0,435	0,565	1,006	0,006
	4,7500	0,324	0,398	0,602	0,925	-0,075
	5,2500	0,294	0,343	0,657	0,952	-0,048
	5,7500	0,294	0,324	0,676	0,970	-0,030
	6,2500	0,235	0,287	0,713	0,948	-0,052
	6,7500	0,206	0,269	0,731	0,937	-0,063
	7,2500	0,176	0,250	0,750	0,926	-0,074
	7,7500	0,176	0,231	0,769	0,945	-0,055
	8,2500	0,176	0,213	0,787	0,964	-0,036
	8,7500	0,147	0,194	0,806	0,953	-0,047
	9,5000	0,147	0,185	0,815	0,962	-0,038
	10,2500	0,147	0,157	0,843	0,990	-0,010
	10,7500	0,118	0,157	0,843	0,960	-0,040
	11,5000	0,088	0,148	0,852	0,940	-0,060
	12,2500	0,059	0,130	0,870	0,929	-0,071
	12,7500	0,059	0,120	0,880	0,938	-0,062
	13,2500	0,059	0,111	0,889	0,948	-0,052
	13,7500	0,059	0,102	0,898	0,957	-0,043
	14,2500	0,059	0,093	0,907	0,966	-0,034
	14,7500	0,059	0,083	0,917	0,975	-0,025
	15,7500	0,029	0,074	0,926	0,955	-0,045
	16,7500	0,029	0,065	0,935	0,965	-0,035
	17,5000	0,029	0,056	0,944	0,974	-0,026
	19,7500	0,029	0,046	0,954	0,983	-0,017
	21,7500	0,029	0,037	0,963	0,992	-0,008
	22,2500	0,029	0,028	0,972	1,002	0,002
	23,0000	0,029	0,019	0,981	1,011	0,011
	24,2500	0,029	0,009	0,991	1,020	0,020
	25,5000	0,000	0,009	0,991	0,991	-0,009
	27,0000	0,000	0,000	1,000	1,000	0,000
stromal FoxP3+ TIL	-1,0000	1,000	1,000	0,000	1,000	0,000
	0,1250	0,882	0,953	0,047	0,929	-0,071
	0,3750	0,853	0,925	0,075	0,928	-0,072
	0,6250	0,794	0,804	0,196	0,990	-0,010
	0,8750	0,765	0,776	0,224	0,989	-0,011
	1,1250	0,735	0,738	0,262	0,997	-0,003
	1,3750	0,676	0,673	0,327	1,004	0,004
	1,6250	0,676	0,636	0,364	1,041	0,041
	1,8750	0,647	0,617	0,383	1,030	0,030
	2,2500	0,559	0,561	0,439	0,998	-0,002
	2,6250	0,529	0,533	0,467	0,997	-0,003
	2,8750	0,529	0,514	0,486	1,015	0,015
	3,2500	0,441	0,486	0,514	0,955	-0,045
	3,6250	0,412	0,477	0,523	0,935	-0,065
	3,8750	0,412	0,467	0,533	0,944	-0,056
	4,5000	0,382	0,411	0,589	0,971	-0,029
	5,5000	0,324	0,299	0,701	1,024	0,024
	6,5000	0,324	0,252	0,748	1,071	0,071
	7,2500	0,324	0,215	0,785	1,109	0,109
	8,0000	0,324	0,168	0,832	1,155	0,155
	8,7500	0,265	0,159	0,841	1,106	0,106
	9,5000	0,265	0,150	0,850	1,115	0,115
	10,5000	0,206	0,121	0,879	1,084	0,084
	11,7500	0,206	0,103	0,897	1,103	0,103
	13,0000	0,147	0,093	0,907	1,054	0,054
	14,2500	0,147	0,084	0,916	1,063	0,063
	15,1250	0,118	0,047	0,953	1,071	0,071
	16,3750	0,118	0,037	0,963	1,080	0,080
	18,0000	0,088	0,037	0,963	1,051	0,051
	19,2500	0,088	0,028	0,972	1,060	0,060
	22,6250	0,029	0,019	0,981	1,011	0,011
	27,6250	0,029	0,009	0,991	1,020	0,020
	32,5000	0,000	0,009	0,991	0,991	-0,009
	36,0000	0,000	0,000	1,000	1,000	0,000
epithelial FoxP3+ TIL	-1,0000	1,000	1,000	0,000	1,000	0,000
	0,2500	0,853	0,860	0,140	0,993	-0,007
	0,7500	0,706	0,766	0,234	0,940	-0,060
	1,2500	0,382	0,439	0,561	0,943	-0,057
	1,7500	0,235	0,252	0,748	0,983	-0,017
	2,2500	0,147	0,159	0,841	0,988	-0,012
	2,7500	0,118	0,075	0,925	1,043	0,043
	3,2500	0,088	0,056	0,944	1,032	0,032
	3,7500	0,088	0,037	0,963	1,051	0,051
	5,0000	0,088	0,019	0,981	1,070	0,070
	6,2500	0,059	0,000	1,000	1,059	0,059
	7,5000	0,029	0,000	1,000	1,029	0,029
	9,5000	0,000	0,000	1,000	1,000	0,000

Supplementary Table 1: Calculation of Youden index by ROC analysis

Exon 9		Exon 11		Exon 14	
c.853T>C	p.F285L	c.1102G>T	p.D368Y	c.1388C>A	p.A463D
c.857C>G	p.P286R			c.1388C>A	p.A463D
c.857C>G	p.P286R				
c.857C>G	p.P286R				
c.857C>G	p.P286R				

Supplementary Table 2: List of POLE mutations

	Infiltration density / absolute values			
	Mean	Median	Minimum	Maximum
overall T-cells (CD3+; % of tumor area)	10.78	6.00	0.00	70.00
epithelial T-cells (CD3+; tumor epithelium; punctum maximum)	6.62	4.28	0.00	60.00
stromal T-cells (CD3+; % of intratumoral stroma)	32.93	28.00	0.00	95.00
overall T-Killer cells (CD8+; % of tumor area)	7.45	3.25	0.00	55.00
epithelial T-Killer cells (CD8+; tumor epithelium; punctum maximum)	5.38	3.75	0.00	26.00
stromal T-Killer cells (CD8+; % of intratumoral stroma)	16.22	7.50	0.00	75.00
overall regulatory T-cells (FoxP3+% of tumor area)	2.38	1.25	0.00	25.00
epithelial regulatory T-cells (FoxP3+; tumor epithelium; punctum maximum)	1.40	1.00	0.00	9.00
stromal regulatory T-cells (FoxP3+; % of intratumoral stroma)	5.14	3.00	0.00	35.00

Supplementary Table 3: Absolute values (mean; median; range) of infiltration densities of tumor infiltrating lymphocytes

		Grading (WHO)						
		G1		G2		G3		
		n	%	n	%	n	%	*p-value*
stromal TIL (CD3)	low	23	16,20%	26	18,30%	14	9,90%	*0.005*
	high	22	15,50%	19	13,40%	38	26,80%	
epithelial TIL (CD3)	low	11	7,70%	15	10,60%	4	2,80%	*0.007*
	high	34	23,90%	30	21,10%	48	33,80%	
stromal T-Killer cells (CD8)	low	18	12,70%	18	12,70%	3	2,10%	*<0.001*
	high	27	19,00%	27	19,00%	49	34,50%	
epithelial T-Killer cells (CD8)	low	23	16,20%	32	22,50%	16	11,30%	*<0.001*
	high	22	15,50%	13	9,20%	36	25,40%	
stromal regulatory T-cells (FoxP3)	low	43	30,30%	38	26,80%	32	22,50%	*<0.001*
	high	2	1,40%	7	4,90%	20	14,10%	
epithelial regulatory T-cells (FoxP3)	low	45	31,70%	44	31,00%	48	33,80%	*0.104*
	high	0	0,00%	1	0,70%	4	2,80%	

		Grading (WHO)				
		low grade (G1/G2)		high grade (G3)		
		n	%	n	%	*p-value*
stromal TIL (CD3)	low	49	34,50%	14	9,90%	*0.001*
	high	41	28,90%	38	26,80%	
epithelial TIL (CD3)	low	26	18,30%	4	2,80%	*0.003*
	high	64	45,10%	48	33,80%	
stromal T-Killer cells (CD8)	low	36	25,40%	3	2,10%	*<0.001*
	high	54	38,00%	49	34,50%	
epithelial T-Killer cells (CD8)	low	55	38,70%	16	11,30%	*<0.001*
	high	35	24,60%	36	25,40%	
stromal regulatory T-cells (FoxP3)	low	81	57,00%	32	22,50%	*<0.001*
	high	9	6,30%	20	14,10%	
epithelial regulatory T-cells (FoxP3)	low	89	62,70%	48	33,80%	*0.004*
	high	1	0,70%	4	2,80%	

		p53 status				
		mutated		wildtype		
		n	%	n	%	*p-value*
stromal TIL (CD3)	low	9	6,30%	54	38,00%	*0.951*
	high	11	7,70%	68	47,90%	
epithelial TIL (CD3)	low	1	0,70%	29	20,40%	*0.057*
	high	19	13,40%	93	65,50%	
stromal T-Killer cells (CD8)	low	6	4,20%	33	23,20%	*0.784*
	high	14	9,90%	89	62,70%	
epithelial T-Killer cells (CD8)	low	11	7,70%	60	42,30%	*0.629*
	high	9	6,30%	62	43,70%	
stromal regulatory T-cells (FoxP3)	low	19	13,40%	94	66,20%	*0.065*
	high	1	0,70%	28	19,70%	
epithelial regulatory T-cells (FoxP3)	low	20	14,10%	117	82,40%	*0.357*
	high	0	0,00%	5	3,50%	

		Microsatellite status				
		MSI		MSS		
		n	%	n	%	*p-value*
stromal TIL (CD3)	low	13	9,20%	50	35,20%	*0.002*
	high	36	25,40%	43	30,30%	
epithelial TIL (CD3)	low	5	3,50%	25	17,60%	*0.021*
	high	44	31,00%	68	47,90%	
stromal T-Killer cells (CD8)	low	10	7,00%	29	20,40%	*0.171*
	high	39	27,50%	64	45,10%	
epithelial T-Killer cells (CD8)	low	20	14,10%	51	35,90%	*0.112*
	high	29	20,40%	42	29,60%	
stromal regulatory T-cells (FoxP3)	low	36	25,40%	77	54,20%	*0.190*
	high	13	9,20%	16	11,30%	
epithelial regulatory T-cells (FoxP3)	low	47	33,10%	90	63,40%	*0.793*
	high	2	1,40%	3	2,10%	

		Microsatellite status						
		MLH1/PMS2 loss		MSH2/MSH6 loss		MSS		
		n	%	n	%	n	%	*p-value*
stromal TIL (CD3)	low	50	35,20%	10	7,00%	3	2,10%	*0.005*
	high	43	30,30%	32	22,50%	4	2,80%	
epithelial TIL (CD3)	low	25	17,60%	4	2,80%	1	0,70%	*0.066*
	high	68	47,90%	38	26,80%	6	4,20%	
stromal T-Killer cells (CD8)	low	29	20,40%	6	4,20%	4	2,80%	*0.025*
	high	64	45,10%	36	25,40%	3	2,10%	
epithelial T-Killer cells (CD8)	low	51	35,90%	15	10,60%	5	3,50%	*0.061*
	high	42	29,60%	27	19,00%	2	1,40%	
stromal regulatory T-cells (FoxP3)	low	77	54,20%	29	20,40%	7	4,90%	*0.072*
	high	16	11,30%	13	9,20%	0	0,00%	
epithelial regulatory T-cells (FoxP3)	low	90	63,40%	40	28,20%	7	4,90%	*0.791*
	high	3	2,10%	2	1,40%	0	0,00%	

		POLE status				
		wildtype		mutated		
		n	%	n	%	*p-value*
stromal TIL (CD3)	low	62	43,70%	1	0,70%	*0.100*
	high	73	51,40%	6	4,20%	
epithelial TIL (CD3)	low	30	21,10%	0	0,00%	*0.160*
	high	105	73,90%	7	4,90%	
stromal T-Killer cells (CD8)	low	37	26,10%	2	1,40%	*0.946*
	high	98	69,00%	5	3,50%	
epithelial T-Killer cells (CD8)	low	70	49,30%	1	0,70%	*0.053*
	high	65	45,80%	6	4,20%	
stromal regulatory T-cells (FoxP3)	low	108	76,10%	5	3,50%	*0.583*
	high	27	19,00%	2	1,40%	
epithelial regulatory T-cells (FoxP3)	low	131	92,30%	6	4,20%	*0.113*
	high	4	2,80%	1	0,70%	

Supplementary Table :4 Correlation of immunologic variables (intraepithelial TIL) with morphomolecular data

		Age				
		median and below		above median		
		n	%	n	%	*p-value*
overall TIL (CD3)	low	47	33,10%	44	31,00%	*0.764*
	high	25	17,60%	26	18,30%	
stromal TIL (CD3)	low	37	26,10%	26	18,30%	*0.088*
	high	35	24,60%	44	31,00%	
epithelial TIL (CD3)	low	16	11,30%	14	9,90%	*0.746*
	high	56	39,40%	56	39,40%	
overall T-Killer cells (CD8)	low	35	24,60%	26	18,30%	*0.168*
	high	37	26,10%	44	31,00%	
stromal T-Killer cells (CD8)	low	26	18,30%	13	9,20%	*0.019*
	high	46	32,40%	57	40,10%	
epithelial T-Killer cells (CD8)	low	37	26,10%	34	23,90%	*0.737*
	high	35	24,60%	36	25,40%	
overall regulatory T-cells (FoxP3)	low	56	39,40%	58	40,80%	*0.447*
	high	16	11,30%	12	8,50%	
stromal regulatory T-cells (FoxP3)	low	56	39,40%	57	40,10%	*0.590*
	high	16	11,30%	13	9,20%	
epithelial regulatory T-cells (FoxP3)	low	70	49,30%	67	47,20%	*0.626*
	high	2	1,40%	3	2,10%	

		T Stage								
		1a/b		2		3		n.a		
		n	%	n	%	n	%	n	%	*p-value*
overall TIL (CD3)	low	68	47,90%	13	9,20%	9	6,30%	1	0,70%	*0.490*
	high	33	23,20%	11	7,70%	7	4,90%	0	0,00%	
stromal TIL (CD3)	low	46	32,40%	10	7,00%	7	4,90%	0	0,00%	*0.819*
	high	55	38,70%	14	9,90%	9	6,30%	1	0,70%	
epithelial TIL (CD3)	low	20	14,10%	6	4,20%	4	2,80%	0	0,00%	*0.865*
	high	81	57,00%	18	12,70%	12	8,50%	1	0,70%	
overall T-Killer cells (CD8)	low	48	33,80%	8	5,60%	5	3,50%	0	0,00%	*0.332*
	high	53	37,30%	16	11,30%	11	7,70%	1	0,70%	
stromal T-Killer cells (CD8)	low	33	23,20%	3	2,10%	3	2,10%	0	0,00%	*0.167*
	high	68	47,90%	21	14,80%	13	9,20%	1	0,70%	
epithelial T-Killer cells (CD8)	low	48	33,80%	13	9,20%	9	6,30%	1	0,70%	*0.645*
	high	53	37,30%	11	7,70%	7	4,90%	0	0,00%	
overall regulatory T-cells (FoxP3)	low	85	59,90%	18	12,70%	10	7,00%	1	0,70%	*0.185*
	high	16	11,30%	6	4,20%	6	4,20%	0	0,00%	
stromal regulatory T-cells (FoxP3)	low	83	58,50%	18	12,70%	11	7,70%	1	0,70%	*0.544*
	high	18	12,70%	6	4,20%	5	3,50%	0	0,00%	
epithelial regulatory T-cells (FoxP3)	low	98	69,00%	23	16,20%	15	10,60%	1	0,70%	*0.917*
	high	3	2,10%	1	0,70%	1	0,70%	0	0,00%	

		N Stage				
		0		1		
		n	%	n	%	*p-value*
overall TIL (CD3)	low	87	61,30%	4	2,80%	*0.100*
	high	45	31,70%	6	4,20%	
stromal TIL (CD3)	low	59	41,50%	4	2,80%	*0.773*
	high	73	51,40%	6	4,20%	
epithelial TIL (CD3)	low	30	21,10%	0	0,00%	*0.090*
	high	102	71,80%	10	7,00%	
overall T-Killer cells (CD8)	low	59	41,50%	2	1,40%	*0.128*
	high	73	51,40%	8	5,60%	
stromal T-Killer cells (CD8)	low	39	27,50%	0	0,00%	*0.044*
	high	93	65,50%	10	7,00%	
epithelial T-Killer cells (CD8)	low	67	47,20%	4	2,80%	*0.512*
	high	65	45,80%	6	4,20%	
overall regulatory T-cells (FoxP3)	low	108	76,10%	6	4,20%	*0.095*
	high	24	16,90%	4	2,80%	
stromal regulatory T-cells (FoxP3)	low	105	73,90%	8	5,60%	*0.973*
	high	27	19,00%	2	1,40%	
epithelial regulatory T-cells (FoxP3)	low	127	89,40%	10	7,00%	*0.531*
	high	5	3,50%	0	0,00%	

		M Stage				
		0		1		
		n	%	n	%	*p-value*
overall TIL (CD3)	low	88	62,00%	3	2,10%	*0.230*
	high	47	33,10%	4	2,80%	
stromal TIL (CD3)	low	60	42,30%	3	2,10%	*0.934*
	high	75	52,80%	4	2,80%	
epithelial TIL (CD3)	low	29	20,40%	1	0,70%	*0.649*
	high	106	74,60%	6	4,20%	
overall T-Killer cells (CD8)	low	59	41,50%	2	1,40%	*0.430*
	high	76	53,50%	5	3,50%	
stromal T-Killer cells (CD8)	low	38	26,80%	1	0,70%	*0.423*
	high	97	68,30%	6	4,20%	
epithelial T-Killer cells (CD8)	low	68	47,90%	3	2,10%	*0.698*
	high	67	47,20%	4	2,80%	
overall regulatory T-cells (FoxP3)	low	110	77,50%	4	2,80%	*0.115*
	high	25	17,60%	3	2,10%	
stromal regulatory T-cells (FoxP3)	low	110	77,50%	3	2,10%	*0.013*
	high	25	17,60%	4	2,80%	
epithelial regulatory T-cells (FoxP3)	low	130	91,50%	7	4,90%	*0.604*
	high	5	3,50%	0	0,00%	

		FIGO Stage								
		I		II		III		IV		
		n	%	n	%	n	%	n	%	*p-value*
overall TIL (CD3)	low	67	47,20%	12	8,50%	9	6,30%	3	2,10%	*0.537*
	high	33	23,20%	7	4,90%	7	4,90%	4	2,80%	
stromal TIL (CD3)	low	45	31,70%	9	6,30%	6	4,20%	3	2,10%	*0.941*
	high	55	38,70%	10	7,00%	10	7,00%	4	2,80%	
epithelial TIL (CD3)	low	20	14,10%	6	4,20%	3	2,10%	1	0,70%	*0.666*
	high	80	56,30%	13	9,20%	13	9,20%	6	4,20%	
overall T-Killer cells (CD8)	low	48	33,80%	7	4,90%	4	2,80%	2	1,40%	*0.259*
	high	52	36,60%	12	8,50%	12	8,50%	5	3,50%	
stromal T-Killer cells (CD8)	low	33	23,20%	3	2,10%	2	1,40%	1	0,70%	*0.155*
	high	67	47,20%	16	11,30%	14	9,90%	6	4,20%	
epithelial T-Killer cells (CD8)	low	48	33,80%	12	8,50%	8	5,60%	3	2,10%	*0.655*
	high	52	36,60%	7	4,90%	8	5,60%	4	2,80%	
overall regulatory T-cells (FoxP3)	low	84	59,20%	15	10,60%	11	7,70%	4	2,80%	*0.203*
	high	16	11,30%	4	2,80%	5	3,50%	3	2,10%	
stromal regulatory T-cells (FoxP3)	low	82	57,70%	14	9,90%	14	9,90%	3	2,10%	*0.066*
	high	18	12,70%	5	3,50%	2	1,40%	4	2,80%	
epithelial regulatory T-cells (FoxP3)	low	97	68,30%	18	12,70%	15	10,60%	7	4,90%	*0.836*
	high	3	2,10%	1	0,70%	1	0,70%	0	0,00%	

		FIGO Stage				
		Low (I / II)		High (III / IV)		
		n	%	n	%	*p-value*
overall TIL (CD3)	low	79	55,60%	12	8,50%	*0.193*
	high	40	28,20%	11	7,70%	
stromal TIL (CD3)	low	54	38,00%	9	6,30%	*0.581*
	high	65	45,80%	14	9,90%	
epithelial TIL (CD3)	low	26	18,30%	4	2,80%	*0.632*
	high	93	65,50%	19	13,40%	
overall T-Killer cells (CD8)	low	55	38,70%	6	4,20%	*0.074*
	high	64	45,10%	17	12,00%	
stromal T-Killer cells (CD8)	low	36	25,40%	3	2,10%	*0.091*
	high	83	58,50%	20	14,10%	
epithelial T-Killer cells (CD8)	low	60	42,30%	11	7,70%	*0.820*
	high	59	41,50%	12	8,50%	
overall regulatory T-cells (FoxP3)	low	99	69,70%	15	10,60%	*0.047*
	high	20	14,10%	8	5,60%	
stromal regulatory T-cells (FoxP3)	low	96	67,60%	17	12,00%	*0.462*
	high	23	16,20%	6	4,20%	
epithelial regulatory T-cells (FoxP3)	low	115	81,00%	22	15,50%	*0.814*
	high	4	2,80%	1	0,70%	

Supplementary Table 5: Correlation of immunologic with clinical data

		Grading (WHO)							Grading (WHO)					p53 status				
		1		2		3			low-grade		high-grade			mutated		wildtype		
		n	%	n	%	n	%	*p-value*	n	%	n	%	*p-value*	n	%	n	%	*p-value*
overall TIL (CD3)	low	30	34,10%	23	26,10%	11	12,50%	*<0,001*	53	60,20%	11	12,50%	*<0,001*	9	10,20%	55	62,50%	*0,849*
	high	3	3,40%	6	6,80%	15	17,00%		9	10,20%	15	17,00%		3	3,40%	21	23,90%	
stromal TIL (CD3)	low	21	23,90%	18	20,50%	10	11,40%	*0,108*	39	44,30%	10	11,40%	*0,035*	5	5,70%	44	50,00%	*0,293*
	high	12	13,60%	11	12,50%	16	18,20%		23	26,10%	16	18,20%		7	8,00%	32	36,40%	
epithelial TIL (CD3)	low	10	11,40%	11	12,50%	4	4,50%	*0,172*	21	23,90%	4	4,50%	*0,079*	0	0,00%	25	28,40%	*0,019*
	high	23	26,10%	18	20,50%	22	25,00%		41	46,60%	22	25,00%		12	13,60%	51	58,00%	
overall T-Killer cells (CD8)	low	23	26,10%	16	18,20%	5	5,70%	*<0,001*	39	44,30%	5	5,70%	*<0,001*	6	6,80%	38	43,20%	*1,000*
	high	10	11,40%	13	14,80%	21	23,90%		23	26,10%	21	23,90%		6	6,80%	38	43,20%	
stromal T-Killer cells (CD8)	low	13	14,80%	13	14,80%	2	2,30%	*0,006*	26	29,50%	2	2,30%	*0,002*	3	3,40%	25	28,40%	*0,585*
	high	20	22,70%	16	18,20%	24	27,30%		36	40,90%	24	27,30%		9	10,20%	51	58,00%	
epithelial T-Killer cells (CD8)	low	16	18,20%	22	25,00%	12	13,60%	*0,04*	38	43,20%	12	13,60%	*0,191*	7	8,00%	43	48,90%	*0,909*
	high	17	19,30%	7	8,00%	14	15,90%		24	27,30%	14	15,90%		5	5,70%	33	37,50%	
overall regulatory T-cells (FoxP3)	low	33	37,50%	24	27,30%	19	21,60%	*0,009*	57	64,80%	19	21,60%	*0,019*	11	12,50%	65	73,90%	*0,565*
	high	0	0,00%	5	5,70%	7	8,00%		5	5,70%	7	8,00%		1	1,10%	11	12,50%	
stromal regulatory T-cells (FoxP3)	low	31	35,20%	24	27,30%	18	20,50%	*0,043*	55	62,50%	18	20,50%	*0,027*	11	12,50%	62	70,50%	*0,388*
	high	2	2,30%	5	5,70%	8	9,10%		7	8,00%	8	9,10%		1	1,10%	14	15,90%	
epithelial regulatory T-cells (FoxP3)	low	33	37,50%	28	31,80%	24	27,30%	*0,271*	61	69,30%	24	27,30%	*0,152*	12	13,60%	73	83,00%	*0,484*
	high	0	0,00%	1	1,10%	2	2,30%		1	1,10%	2	2,30%		0	0,00%	3	3,40%	

Supplementary Table 6: Correlation of immunologic data with morphomolecular data in the subgroup of POLE wildtype and microsatellite stable (MSS) endometrial carcinomas

		Overall	Events (OS)	Events (DSS)	Events (DFS)
overall TIL (CD3)	low	91	30	18	22
	high	51	24	16	16
stromal TIL (CD3)	low	63	19	12	16
	high	79	35	22	22
epithelial TIL (CD3)	low	30	8	4	8
	high	112	46	30	30
overall T-Killer cells (CD8)	low	61	18	9	11
	high	81	36	25	27
stromal T-Killer cells (CD8)	low	39	9	5	6
	high	103	45	29	32
epithelial T-Killer cells (CD8)	low	71	26	19	23
	high	71	28	15	15
overall regulatory T-cells (FoxP3)	low	114	41	25	27
	high	28	13	9	11
stromal regulatory T-cells (FoxP3)	low	113	40	23	26
	high	29	14	11	12
epithelial regulatory T-cells (FoxP3)	low	137	50	31	26
	high	5	4	3	2

Supplementary Table 7: Contexture of immunologic tumor microenvironment and survival associations

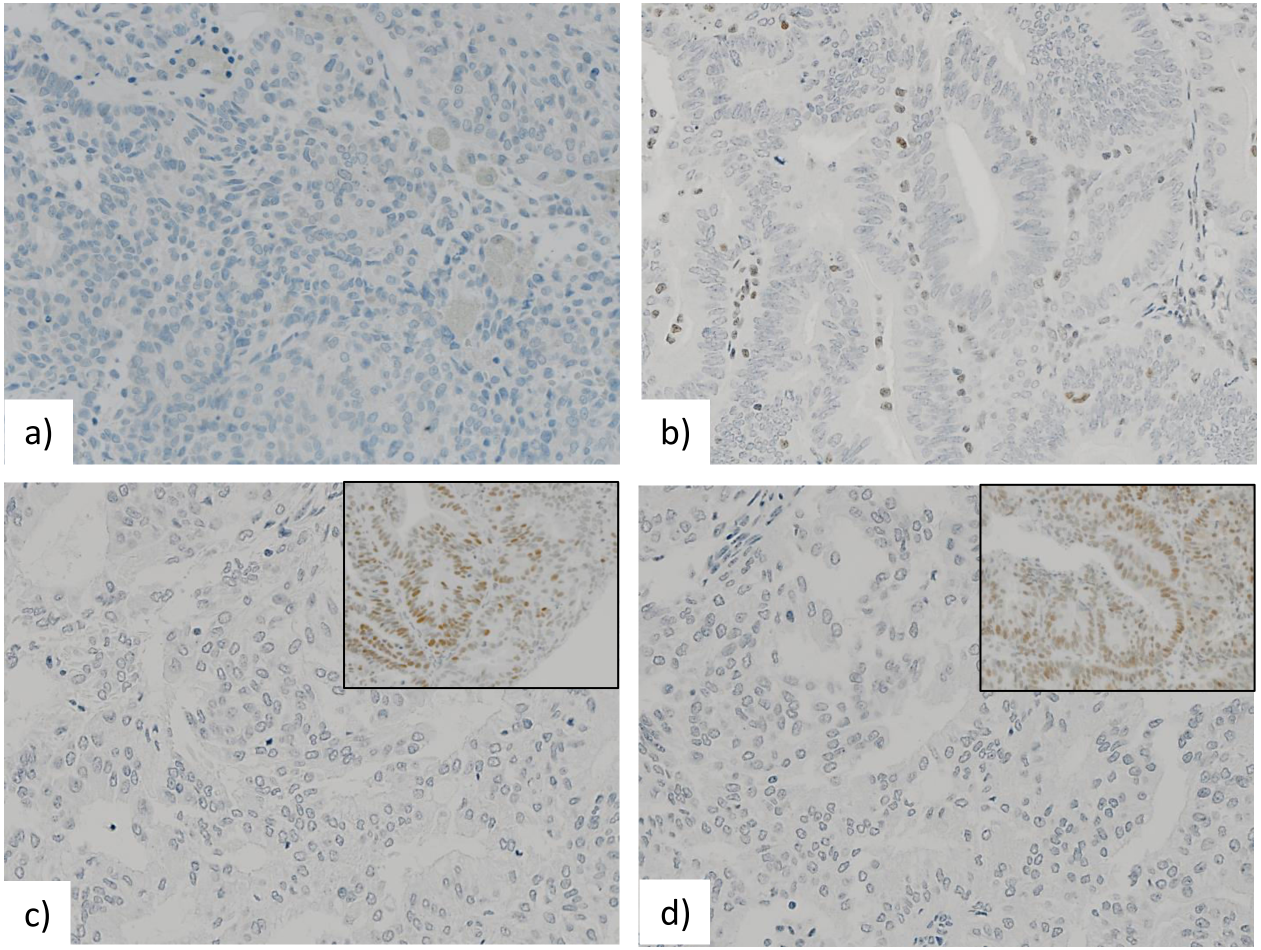
a)
b)
c)
d)

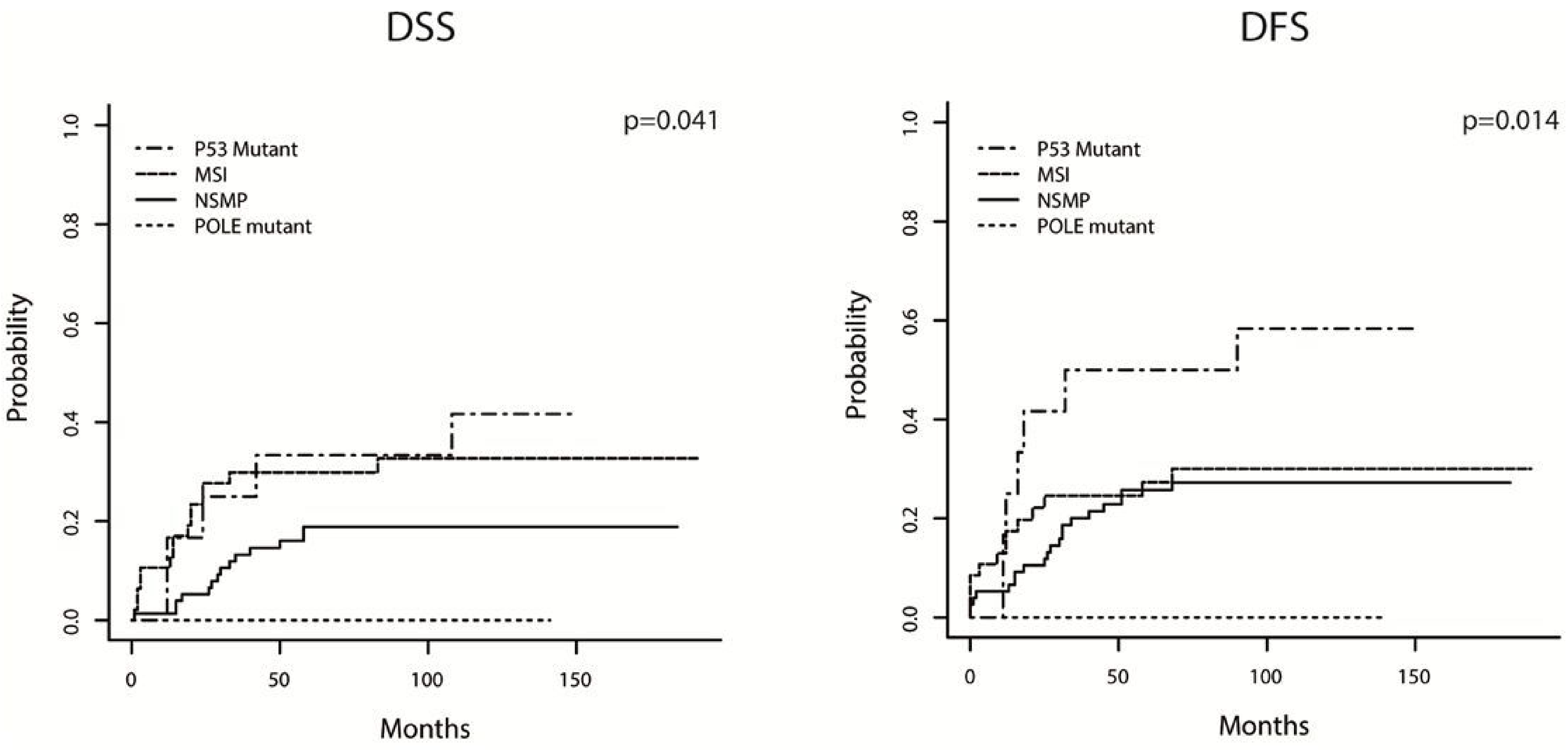

Supplementary Figure 2: Cumulative incidence function visualizing disease-specific and disease-free survival depending on molecular subgroups; a) disease-specific survival; b) disease-free survival.
